# Real-world Effectiveness of Molnupiravir and Nirmatrelvir/Ritonavir as Treatments for COVID-19 in Patients at High Risk

**DOI:** 10.1093/infdis/jiad324

**Published:** 2023-08-11

**Authors:** Dimitrios Paraskevis, Maria Gkova, Kassiani Mellou, Gerasimos Gerolymatos, Naya Psalida, Kassiani Gkolfinopoulou, Evangelia-Georgia Kostaki, Stylianos Loukides, Anastasia Kotanidou, Athanasios Skoutelis, Eleftherios Thiraios, Georgios Saroglou, Dimitrios Zografopoulos, Dimitrios Filippou, Elias Mossialos, Theoklis Zaoutis, Mina Gaga, Sotirios Tsiodras, Anastasia Antoniadou

**Affiliations:** Department of Hygiene, Epidemiology, and Medical Statistics, Medical School, National and Kapodistrian University of Athens; National Public Health Organization; National Public Health Organization; National Public Health Organization; National Public Health Organization; National Public Health Organization; National Public Health Organization; Department of Hygiene, Epidemiology, and Medical Statistics, Medical School, National and Kapodistrian University of Athens; Second Respiratory Medicine Department, “Attikon” University Hospital, Medical School, National and Kapodistrian University of Athens; First Department of Critical Care Medicine and Pulmonary Services, Evaggelismos Hospital, Medical School, National and Kapodistrian University of Athens; Second Department of Medicine and Infectious Diseases, HYGEIA Hospital, Marousi; National Agency for Quality Assurance in Health, Athens; Department of Internal Medicine, Metropolitan Hospital; Greek Ministry of Health; Department of Anatomy and Surgical Anatomy, Medical School, National and Kapodistrian University of Athens; National Organization for Medicines, Athens, Greece; Department of Health Policy, London School of Economics and Political Science; Institute of Global Health Innovation, Imperial College London, United Kingdom; National Public Health Organization; Infectious Diseases Unit, Second Department of Pediatrics, “P. and A. Kyriakou” Children's Hospital, National and Kapodistrian University of Athens; Seventh Respiratory Medicine Department, General Hospital for Chest Diseases of Athens “SOTIRIA,”; National Public Health Organization; Fourth Department of Internal Medicine, National and Kapodistrian University of Athens, Attikon Hospital, Athens, Greece; Fourth Department of Internal Medicine, National and Kapodistrian University of Athens, Attikon Hospital, Athens, Greece

**Keywords:** antivirals, COVID-19, molnupiravir, nirmatrelvir/ritonavir, SARS-CoV-2

## Abstract

**Background:**

Using a retrospective cohort study design, we aimed to evaluate the effectiveness of molnupiravir and nirmatrelvir/ritonavir in patients with SARS-CoV-2 who were highly vulnerable.

**Methods:**

The impact of each drug was determined via comparisons with age-matched control groups of patients positive for SARS-CoV-2 who did not receive oral antiviral therapy.

**Results:**

Administration of molnupiravir significantly reduced the risk of hospitalization (odds ratio [OR], 0.40; *P* < .001) and death (OR, 0.31; *P* < .001) among these patients based on data adjusted for age, previous SARS-CoV-2 infection, vaccination status, and time elapsed since the most recent vaccination. The reductions in risk were most profound among elderly patients (≥75 years old) and among those with high levels of drug adherence. Administration of nirmatrelvir/ritonavir also resulted in significant reductions in the risk of hospitalization (OR, 0.31; *P* < .001) and death (OR, 0.28; *P* < .001). Similar to molnupiravir, the impact of nirmatrelvir/ritonavir was more substantial among elderly patients and in those with high levels of drug adherence.

**Conclusions:**

Collectively, these real-world findings suggest that although the risks of hospitalization and death due to COVID-19 have been reduced, antivirals can provide additional benefits to members of highly vulnerable patient populations.

## Teaser

Molnupiravir significantly reduced the risk of hospitalization (odds ratio [OR], 0.40) and death (OR, 0.31) among patients aged ≥65 years. Nirmatrelvir/ritonavir use similarly resulted in significant reductions in the risk of hospitalization (OR, 0.31) and death (OR, 0.28).

Coronavirus disease 2019 (COVID-19) is caused by SARS-CoV-2 infection, which in most individuals is mild to moderate; however, older people and those with chronic diseases are at higher risk to develop severe disease. Although primary and boosted vaccination against SARS-CoV-2 has significantly reduced the risk of severe disease and death [1–4], COVID-19 remains a significant problem for highly vulnerable populations, including the elderly and those who are immunocompromised or have chronic diseases [5–7]. Thus, the availability of oral antiviral agents that are effective against SARS-CoV-2 might further reduce the risk of hospitalization, disease progression, and death, particularly among patients at high risk. Remdesivir, which was the first antiviral agent approved for emergency treatment of COVID-19, has been shown to prevent disease progression in an outpatient setting, as well as in hospitalized patients with moderate to severe disease [8, 9]. Nirmatrelvir/ritonavir and molnupiravir are newer drugs that also exhibit antiviral activity against SARS-CoV-2 in vitro and in vivo. However, while remdesivir is an intravenous drug, molnupiravir and nirmatrelvir/ritonavir can be administered orally, which is advantageous in an outpatient setting.

In a randomized, double-blind, phase 3 clinical trial (MOVe-OUT), outpatient use of molnupiravir was shown to reduce the risk of hospitalization by 30% as compared with placebo [10]. Similarly, nirmatrelvir/ritonavir significantly reduced the risk of hospitalization and death (relative risk [RR] reduction, 88.9%) for at least 28 days after administration in patients with COVID-19 who were symptomatic, unvaccinated, and nonhospitalized and at high risk for progression to severe disease [11]. Nirmatrelvir/ritonavir ultimately received emergency use authorization from the Food and Drug Administration in January 2022 for the treatment of COVID-19 in patients who were at high risk for severe disease but did not require supplemental oxygen [12]. While outpatient administration of molnupiravir or nirmatrelvir/ritonavir in patients who were diagnosed with COVID-19 and at high risk for disease progression has resulted in a significant reduction in the risk of hospitalization and death in real-world studies [13–19], critical questions remain.

In this study, we aimed to explore the real-world effectiveness of molnupiravir and nirmatrelvir/ritonavir administered to outpatients diagnosed with COVID-19 who were at high risk of developing severe disease. We evaluated outcomes that included (1) hospital admission without transfer to the intensive care unit (ICU), clinical deterioration, or death and (2) death due to COVID-19. The effectiveness of these antivirals was explored by comparing the outcomes documented for recipients of molnupiravir and nirmatrelvir/ritonavir with age-matched nonrecipient controls.

## METHODS

### Study Population

Our study population consisted of all nonhospitalized patients ≥65 years old who were infected with SARS-CoV-2 and received either of the following:

- Molnupiravir (800 mg; 4 capsules) every 12 hours for 5 days between 2 February 2022 and 5 March 2022
- Nirmatrelvir/ritonavir (300 mg/100 mg; 3 tablets) every 12 hours for 5 days between 26 March 2022 and 20 July 2022

The latter dose was reduced to nirmatrelvir 150 mg and ritonavir 100 mg in patients who presented with an estimated glomerular filtration rate of 30 to 59 mL/min/1.73 m².

Specifically, among the total population in Greece treated with molnupiravir (n = 6474) and nirmatrelvir/ritonavir (n = 23 191), 65.5% (n = 4240) and 59.8% (n = 13 861) were respectively identified as outpatient recipients aged ≥65 years and therefore included in the study. We selected only the group ≥65 years old, since comorbidities were unknown among the nonrecipients (controls). Given that comorbidities increase with age, age-matched controls are very likely to have a similar pattern of comorbidities as the antiviral recipients.

Further information about the study population and design is provided in the supplementary data.

### Outcomes

The main outcomes of interest included (1) hospitalization for COVID-19 within 10 days after a positive SARS-CoV-2 test result with no ICU admission or clinical deterioration (intubation); (2) death from COVID-19 within 35 days following a positive SARS-CoV-2 test result; and (3) hospitalization, ICU admission, intubation, or death from COVID-19 within 35 days following a positive SARS-CoV-2 test result.

### Ethical Approval

The study was approved by the Data Protection Officer of the Ministry of Health and by the Ethical Committee of the National Public Health Organization in Greece.

### Statistical Analysis

Factors associated with various outcomes were estimated by multivariable logistic regression analysis: hospitalization, COVID-19–associated death, or combined outcomes associated with hospital admission (including ICU admission, clinical deterioration, or death). This method was used to identify the RR of each drug (odds ratio [OR]) with respect to symptomatic disease, death, or the combined outcome in patients treated with oral antivirals vs nonrecipient controls, adjusted for age, previous SARS-CoV-2 infection, vaccination status, and time elapsed since the most recent vaccination. Additional information is shown in supplementary data.

## Results

To evaluate the real-life effectiveness of molnupiravir, we identified 4240 eligible recipients from outpatient services data with 4240 nonrecipient controls. These cohorts were matched for age and the calendar period during which they contracted SARS-CoV-2 infection. While 6470 molnupiravir recipients were included in the database overall, we limited our cohort to those who were ≥65 years old (65% of the total). Because we have information on comorbidities only for patients who received treatment, we decided to focus on patients in this older cohort. Given the high prevalence of comorbidities in this age group, we are substantially less likely to introduce selection bias with respect to the frequency of comorbidities in age-matched controls. The characteristics of the study population are shown in Table 1 and described in detail in the supplementary data.

**Table 1.** Characteristics of the Study Population

	Molnupiravir				Νirmatrelvir/Ritonavir			
	Recipients		Nonrecipients		Recipients		Nonrecipients	
	No.	%	No.	%	No.	%	No.	%
Outcome								
No hospitalization, clinical deterioration, or COVID-19-associated deaths	4022	94.86	3725	87.85	13 462	97.12	12 728	91.83
Hospitalization without ICU admission, clinical deterioration, or death	149	3.51	357	8.42	297	2.14	857	6.18
ICU admission or clinical deterioration	18	0.42	14	0.33	14	0.10	17	0.12
COVID-19–associated death	51	1.20	144	3.40	88	0.63	259	1.87
Hospitalization, ICU admission, clinical deterioration, or death	218	5.14	515	12.14	399	2.88	1133	8.17
Vaccination status								
Unvaccinated	534	12.6	859	20.3	1312	9.5	2074	15.0
Single dose	61	1.4	62	1.5	53	0.4	37	0.3
Two doses: before index SARS-CoV-2 infection								
≤6 mo	220	5.2	426	10.0	458	3.3	657	4.7
>6 mo	66	1.6	85	2.0	294	2.1	412	3.0
First booster: before index SARS-CoV-2 infection								
≤6 mo	3318	78.3	2800	66.0	3533	25.5	3745	27.0
>6 mo	6	0.1	2	0.0	5906	42.6	5681	41.0
Second booster	35	0.8	6	0.1	2305	16.6	1255	9.1
Gender								
Male	2128	50.2	2129	50.2	6671	48.1	6462	46.6
Female	2112	49.8	2111	49.8	7190	51.9	7399	53.4
Age, y								
65–69	787	18.6	787	18.6	2775	20.0	2775	20.0
70–74	903	21.3	903	21.3	2958	21.3	2958	21.3
75–79	817	19.3	817	19.3	2930	21.1	2930	21.1
≥80	1733	40.9	1733	40.9	5198	37.5	5198	37.5

Abbreviation: ICU, intensive care unit.

The effectiveness of molnupiravir was evaluated by 3 main outcomes: (1) symptomatic disease that did not lead to death (ie, hospitalization without ICU admission, clinical deterioration, or death; (2) death due to COVID-19; and (3) hospitalization, ICU admission, clinical deterioration, or death. Outcomes determined for recipients of molnupiravir and nonrecipients are shown in Table 1. Among the recipients, 3.51% (n = 149), 0.42% (n = 18), and 1.20% (n = 51) presented with severe symptomatic disease (ICU admission or clinical deterioration without death) or death due to COVID-19, respectively. By contrast, 8.42% of the age-matched nonrecipients developed symptomatic disease (n = 357) and 3.40% died (n = 144). The comorbidities exhibited by these patients are shown in Table 2 and described in the supplementary data.

**Table 2.** Comorbidities and Treatment Adherence

	Molnupiravir Recipients		Νirmatrelvir/Ritonavir Recipients	
	No.	%	No.	%
Comorbidity				
Obesity: BMI ≥35 kg/m^2^	435	10.26	2250	16.23
Cardiovascular disease	1964	46.32	9668	69.75
Moderate-severe immunosuppression	323	7.62	1209	8.72
Type 2 diabetes mellitus	808	19.06	3827	27.61
Chronic disease				
Liver	16	0.38	51	0.37
Kidney	239	5.64	598	4.31
Lung	201	4.74	1046	7.55
Treatment adherence: No. of pills missed				
None	3207	75.7	2626	19.0
0–5	353	8.3	308	2.2
>5 to 12	92	2.2	141	1.0
>12 but not all	172	4.1	117	0.8
All	416	9.8	309	2.2
Unknown	0	0.0	10 360	74.7

Abbreviation: BMI, body mass index.

For the second part of the study, we identified 13 861 eligible recipients of nirmatrelvir/ritonavir and 13 861 matched nonrecipient controls. Our study enrolled those who were ≥65 years old, representing 93.9% of the total population receiving nirmatrelvir/ritonavir (n = 14 718). The characteristics of the study population are shown in Table 1. Similar to the patients who were treated with molnupiravir, many of those taking nirmatrelvir/ritonavir were ≥80 years old (37.5%) and had undergone the basic vaccination scheme (90.2%**;**  supplementary data).

The comorbidities exhibited by the recipients are listed in Table 2. Those appearing most frequently were cardiovascular disease (69.8%), type 2 diabetes mellitus (27.6%), and obesity (body mass index ≥35 kg/m^2^; 16.2%). Self-reported data on nirmatrelvir/ritonavir drug adherence were available for 25.6% (n = 3501) of the population. An overall 75% of those who received nirmatrelvir/ritonavir reported missing no pills (ie, complete adherence); 8.8% and 4.0% missed ≤5 and ≤12 pills, respectively; and 3.3% and 8.8% missed >12 and all pills **(**partial/poor adherence).

Information on adverse drug reactions was available for 3274 recipients (Supplementary Table 1). Of these, 5.4% had ≥1 adverse drug reactions, while 94.7% had none. Gastrointestinal effects, allergy, headache/dizziness, and other events were reported by 1.03%, 0.02%, 0.08%, and 0.20% of the population.

The effectiveness of molnupiravir and nirmatrelvir/ritonavir at preventing hospitalization or death due to COVID-19 was estimated after comparing the outcomes between the recipients and nonrecipients separately for each drug. Effectiveness was estimated for the 3 most frequent outcomes: (1) hospitalization without ICU admission, clinical deterioration, or death; (2) death due to COVID-19; and (3) hospitalization, ICU admission, clinical deterioration, or death due to COVID-19 within 35 days following a positive SARS-CoV-2 test result.

The results of multivariable logistic regression analyses for molnupiravir are presented in detail in Table 3. Statistical analysis revealed that the RR for symptomatic disease (model 1: OR, 0.40; *P* < .001) or death (model 2: OR, 0.31; *P* < 001) was significantly lower for the recipients as compared with nonrecipients. The RR for hospitalization was lower among recently vaccinated individuals (≤6 months previously; OR, 0.36; *P* < .001) and for those reporting a previous SARS-CoV-2 infection (OR, 0.45; *P* = .05). By contrast, this risk increased with patient age (OR, 1.06 per year; *P* < .001). The drug had no impact on those who had not been recently vaccinated, most likely due to the small number **i**n this group. Similar results were obtained for death (model 2), with lower RRs for death observed among those who had been recently vaccinated (OR, 0.36; *P* < .001); higher RRs were observed for older individuals (OR, 1.11 per year; *P* < .001). No effect was observed among those who reported a previous SARS-CoV-2 infection or were vaccinated **>**6 months before the acute event (model 2).

**Table 3.** Multivariable Logistic Regression Analysis to Determine the Effectiveness of Molnupiravir

Explanatory Variable	Odds Ratio	95% CI	*P* Value
Model 1^a^			
Treatment			
Nonrecipients	1.00	…	…
Molnupiravir recipients	0.40	.32–.48	<.001
Age, per year	1.06	1.04–1.07	<.001
Previous COVID-19			
No previous SARS-CoV-2 infection	1.00	…	…
Previous SARS-CoV-2 infection	0.45	.19–.92	.05
Vaccination status			
Unvaccinated	1.00	…	…
Vaccination: 2, 3, or 4 doses ≤6 mo before index SARS-CoV-2 infection	0.36	.30–.45	<.001
Vaccination: 2, 3, or 4 doses >6 mo before index SARS-CoV-2 infection	1.08	.63–1.76	.772
Model 2^b^			
Treatment			
Nonrecipients	1.00	…	…
Molnupiravir recipients	0. 31	.22–.43	<.001
Age, per year	1.11	1.09–1.14	<.001
Previous COVID-19			
No previous SARS-CoV-2 infection	1.00	…	…
Previous SARS-CoV-2 infection	0.56	.13–1.57	.34
Vaccination status			
Unvaccinated	1.00	…	…
Vaccination: 2, 3, or 4 doses ≤6 mo before index SARS-CoV-2 infection	0.36	.26–.49	<.001
Vaccination: 2, 3, or 4 doses >6 mo before index SARS-CoV-2 infection	1.22	.55–2.45	.60

Abbreviation: ICU, intensive care unit.

^a^Model 1: hospitalization without ICU admission, clinical deterioration, or death vs no hospitalization, ICU admission, clinical deterioration, or death.

^b^Model 2: death with or without ICU admission or clinical deterioration vs no hospitalization, ICU admission, clinical deterioration, or death.

The RR for the combined outcome of hospitalization, ICU admission, clinical deterioration, and death was similar to hospitalization alone (OR, 0.40 [*P* < .001]; model 1, Supplementary Table 2), although recent vaccination, patient age, and previous infection also had an effect. To determine whether the treatment effect was age dependent, we performed analysis of symptomatic disease by age group (models 2–5). This analysis revealed that molnupiravir was most effective in older patients, specifically those aged 75 to 79 and ≥80 years. The RR of severe disease decreased with age in members of the treatment group (models 3b, 4, and 5).

When we considered treatment adherence in the model limited to patients receiving molnupiravir, we found that those reporting complete adherence had a significantly lower risk for hospitalization (OR, 0.27; *P* < .001) as compared with those reporting poor or incomplete adherence (model 1, Supplementary Table 3). A similar effect was observed for COVID-19–associated death (OR, 0.24 [*P* < .001]; model 2). Collectively, these results suggest that molnupiravir was approximately 70% more effective in patients who were fully adherent vs those reporting poor or only partial adherence to this drug regimen.

The results of multivariable logistic regression analyses for nirmatrelvir/ritonavir are presented in detail in Table 4. The RR for symptomatic disease or death (models 1 and 2, Supplementary Table 3) was significantly lower for those receiving antiviral treatment (model 1: OR, 0.31 [*P* < .001]; model 2: OR, 0.28 [*P* < .001]). The RR for hospitalization was lower for those who reported a previous SARS-CoV-2 infection (OR, 0.55; *P* = .003), were recently vaccinated (OR, 0.53; *P* < .001), or were vaccinated >6 months earlier (OR, 0.49; *P* < .001).

**Table 4.** Multivariable Logistic Regression Analysis to Determine the Effectiveness of Nirmatrelvir/Ritonavir Treatment

Explanatory Variable	Odds Ratio	95% CI	*P* Value
Model 1^a^			
Treatment			
Nonrecipients	1.00	…	…
Nirmatrelvir/ritonavir recipients	0.31	.27–.36	<.001
Age, per year	1.09	1.08–1.10	<.001
Previous COVID-19			
No previous SARS-CoV-2 infection	1.00	…	…
Previous SARS-CoV-2 infection	0.55	.37–.80	.003
Vaccination status			
Unvaccinated	1.00	…	…
Vaccination: 2, 3, or 4 doses ≤6 mo before index SARS-CoV-2 infection	0.53	.44–.63	<.001
Vaccination: 2, 3, or 4 doses >6 mo before index SARS-CoV-2 infection	0.49	.41–.59	<.001
Model 2^b^			
Treatment			
Nonrecipients	1.00	…	…
Nirmatrelvir/ritonavir recipients	0.28	.22–.36	<.001
Age, per year	1.16	1.15–1.18	<.001
Previous COVID-19			
No previous SARS-CoV-2 infection	1.00	…	…
Previous SARS-CoV-2 infection	0.32	.14–.64	.003
Vaccination status			
Unvaccinated	1.00	…	…
Vaccination: 2, 3, or 4 doses ≤6 mo before index SARS-CoV-2 infection	0.42	.32–.55	<.001
Vaccination: 2, 3, or 4 doses >6 mo before index SARS-CoV-2 infection	0.25	.18–.34	<.001

Abbreviation: ICU, intensive care unit.

^a^Model 1: hospitalization without ICU admission, clinical deterioration, or death vs no hospitalization, ICU admission, clinical deterioration, or death.

^b^Model 2: death with or without ICU admission or clinical deterioration vs no hospitalization, ICU admission, clinical deterioration, or death.

By contrast, the RR for developing symptomatic disease increased with age (OR, 1.09 per year [*P* < .001]; model 1, Table 4). The RR of death was significantly lower for patients receiving antiviral treatment (OR, 0.28; *P* < .001), those reporting a previous SARS-CoV-2 infection (OR, 0.32; *P* = .003), those recently vaccinated (OR, 0.42; *P* < .001), and those vaccinated >6 months earlier (OR, 0.25 [*P* < .001]; model 2). By contrast, the RR of death increased with age (OR, 1.16 per year [*P* < .001]; model 2).

The RR for the combined outcome of hospitalization, ICU admission, or death was similar to that shown in model 1 (OR, 0.32 [*P* < .001]; model 1, Supplementary Table 3 and Table 4). The results of our age-specific analysis are shown in Supplementary Table 4 (models 2–5).

Patients reporting complete adherence to the nirmatrelvir/ritonavir regimen had a significantly lower risk of hospitalization (OR, 0.27; *P* < .001) as compared with those reporting poor or incomplete adherence (Supplementary Table 5, model 1). Similar results were identified for the risk of death (OR, 0.25 [*P* = .01]; model 2).

To identify potential differences between the antiviral treatment groups, as well as the effect of comorbidities, we estimated the relative effectiveness of nirmatrelvir/ritonavir vs molnupiravir (Table 5). Only the patients who received antiviral treatment were included in this analysis. The results of this are presented in the supplementary data.

**Table 5.** Multivariable Logistic Regression Analysis to Compare Treatment Effectiveness in Molnupiravir and Nirmatrelvir/Ritonavir Recipients

Explanatory Variable	Odds Ratio	95% CI	*P* Value
Model 1^a^			
Treatment			
Molnupiravir recipients	1.00	…	…
Nirmatrelvir/ritonavir recipients	0.58	.46–.75	<.001
Age, per year	1.05	1.03–1.06	<.001
Previous COVID-19			
No previous SARS-CoV-2 infection	1.00	…	…
Previous SARS-CoV-2 infection	0.25	.09–.56	.003
Vaccination status			
Nonvaccinated	1.00	…	…
Vaccination: 2, 3, or 4 doses ≤6 mo before index SARS-CoV-2 infection	0.32	.25–.43	<.001
Vaccination: 2, 3, or 4 doses >6 mo before index SARS-CoV-2 infection	0.43	.32–.59	<.001
Comorbidities^b^			
None	1.00	…	…
1	1.15	.85–1.56	.37
2	1.56	1.12–2.17	.008
3	2.05	1.30–3.18	.002
>3	2.56	.97–5.65	.03
Model 2^c^			
Treatment			
Molnupiravir recipients	1.00	…	…
Nirmatrelvir/ritonavir recipients	0.69	.46–1.06	.09
Age, per year	1.11	1.09–1.14	<.001
Previous COVID-19			
No previous SARS-CoV-2 infection	1.00	…	…
Previous SARS-CoV-2 infection	0.30	.48–1.02	.10
Vaccination status			
Nonvaccinated	1.00	…	…
Vaccination: 2, 3, or 4 doses ≤6 mo before index SARS-CoV-2 infection	0.29	.19–.46	<.001
Vaccination: 2, 3, or 4 doses >6 mo before index SARS-CoV-2 infection	0.22	.12–.40	<.001
Comorbidities			
None	1.00	…	…
1	0.70	.44–1.14	.14
2	0.85	.49–1.48	.57
3	1.08	.43–2.38	.86
>3	4.82	1.58–12.04	.002
Model 3^d^			
Treatment			
Molnupiravir recipients	1.00	…	…
Nirmatrelvir/ritonavir recipients	0.30	.16–.56	<.001
Previous iCOVID-19			
No previous SARS-CoV-2 infection	1.00	…	…
Previous SARS-CoV-2 infection	0.50	.08–1.91	.38
Vaccination status			
Nonvaccinated	1.00	…	…
Vaccination: 2, 3, or 4 doses ≤6 mo before index SARS-CoV-2 infection	0.32	.16–.67	.001
Vaccination: 2, 3, or 4 doses >6 mo before index SARS-CoV-2 infection	0.45	.19–1.09	.07
Model 4^e^			
Treatment			
Molnupiravir recipients	1.00	…	…
Nirmatrelvir/ritonavir recipients	0.56	.32–.98	.04
Previous COVID-19			
No previous SARS-CoV-2 infection	1.00	…	…
Previous SARS-CoV-2 infection	0.29	.02–1.51	.24
Vaccination status			
Nonvaccinated	1.00	…	…
Vaccination: 2, 3, or 4 doses ≤6 mo before index SARS-CoV-2 infection	0.45	.23–.93	.02
Vaccination: 2, 3, or 4 doses >6 mo before index SARS-CoV-2 infection	0.54	.26–1.21	.12
Model 5^f^			
Treatment			
Molnupiravir recipients	1.00	…	…
Nirmatrelvir/ritonavir recipients	0.67	.40–1.16	.15
Previous COVID-19			
No previous SARS-CoV-2 infection	1.00	…	…
Previous SARS-CoV-2 infection	1.20	.32–3.64	.76
Vaccination status			
Nonvaccinated	1.00	…	…
Vaccination: 2, 3, or 4 doses ≤6 mo before index SARS-CoV-2 infection	0.44	.23–.91	.02
Vaccination: 2, 3, or 4 doses >6 mo before index SARS-CoV-2 infection	0.34	.16–.76	.007
Model 6^g^			
Treatment			
Molnupiravir recipients	1.00	…	…
Nirmatrelvir/ritonavir recipients	0.69	.50–.97	.03
Vaccination status			
Nonvaccinated	1.00	…	…
Vaccination: 2, 3, or 4 doses ≤6 mo before index SARS-CoV-2 infection	0.29	.21–.97	<.001
Vaccination: 2, 3, or 4 doses >6 mo before index SARS-CoV-2 infection	0.42	.28–.64	<.001
Comorbidities			
None	1.00	…	…
1	1.27	.87–1.91	.22
2	1.52	.99–2.36	.06
3	1.98	1.06–3.56	.03
>3	2.05	.48–5.97	.25

Abbreviations: BMI, body mass index; ICU, intensive care unit.

Model 1: hospitalization without ICU admission, clinical deterioration, or death vs no hospitalization, ICU admission, clinical deterioration, or death.

Comorbidities include obesity (BMI ≥35 kg/m^2^), cardiovascular disease, T2DM, chronic liver disease, chronic kidney disease, and chronic lung disease. Individuals diagnosed with moderate to severe immunosuppression were excluded.

Model 2: death with or without ICU admission or clinical deterioration vs no hospitalization, ICU admission, clinical deterioration, or death.

Model 3: hospitalization without ICU admission, clinical deterioration, or death vs no hospitalization, ICU admission, clinical deterioration, or death among those 65 to 69 years of age.

Model 4: hospitalization without ICU admission, clinical deterioration, or death vs no hospitalization, ICU admission, clinical deterioration, or death among those 70 to 74 years of age.

Model 5: hospitalization without ICU admission, clinical deterioration, or death vs no hospitalization, ICU admission, clinical deterioration, or death among those 75 to 79 years of age.

Model 6: hospitalization without ICU admission, clinical deterioration, or death versus no hospitalization, ICU admission, clinical deterioration, or death among those ≥80 y of age.

## DISCUSSION

Our study documents the real-world effectiveness of oral antivirals directed against SARS-CoV-2 in outpatients at high risk of disease progression. The analysis included all eligible recipients of molnupiravir and nirmatrelvir/ritonavir who were ≥65 years old and registered in a public database in Greece. Our findings revealed that the administration of molnupiravir or nirmatrelvir/ritonavir reduced the risk of hospitalization and death in this highly vulnerable patient cohort. Specifically, the risk of hospital admission was significantly lower for molnupiravir recipients, most notably among those who were ≥75 years old. Of note, the estimated RR for hospitalization was adjusted for vaccination status and the time elapsed since the most recent previous vaccination. These results suggest that molnupiravir treatment is associated with a reduced risk for severe disease independent of vaccination. Given that most study participants had received a first booster dose within 6 months of the index SARS-CoV-2 infection, our results indicate that (1) molnupiravir treatment reduces the risk of hospitalization even among recent vaccinees and those who have received a third dose and (2) the treatment effect was more pronounced among the oldest members of the study population. Molnupiravir treatment also resulted in a reduction in the adjusted risk for death from COVID-19 as compared with nonrecipients among those aged ≥65 years. Molnupiravir recipients who reported complete adherence to the drug regimen exhibited significantly lower rates of hospitalization or death vs patients who reported poor or incomplete adherence. Collectively, these results suggest that patients must adhere to the prescribed drug regimen to benefit from these positive effects.

We observed a similarly reduced risk of hospital admission or death from COVID-19 among nirmatrelvir/ritonavir recipients as compared with nonrecipients, independent of the vaccination status, the time elapsed since the last vaccination, and previous SARS-CoV-2 infection. The impact of nirmatrelvir/ritonavir was also most significant in the older groups, most notably those ≥70 years old. Interestingly, among those who received nirmatrelvir/ritonavir, a longer interval between the last single booster was associated with a reduced OR of hospitalization or death, in contrast to our findings for the molnupiravir recipients. Considering that a much higher proportion of patients who received nirmatrelvir/ritonavir had been vaccinated within 6 months of their infection, the lack of protection observed among molnupiravir recipients with prolonged intervals between vaccination and SARS-CoV-2 infection may be attributed to the small number of patients in this subgroup. Similar to what we observed in response to treatment with molnupiravir, patients who reported complete adherence to nirmatrelvir/ritonavir had a reduced risk for hospitalization and death as compared with those who reported poor or incomplete compliance.

A comparative analysis of the responses to these drugs revealed that nirmatrelvir/ritonavir recipients exhibited a lower risk of hospitalization and death when compared with those treated with molnupiravir. The differences observed were based largely on the lower incidence of death secondary to COVID-19 vs hospital admission. Notably, nirmatrelvir/ritonavir recipients exhibited a reduced risk for hospitalization vs molnupiravir recipients who were in the youngest age cohort (65–69 years); the differences in the risk were smaller among those aged 70 to 74 and ≥80 years. The risk of hospitalization or death increased in parallel with the number of comorbidities as compared with those who reported no health conditions. These findings suggest that the effectiveness of nirmatrelvir/ritonavir was higher than molnupiravir and that the difference was more pronounced in the younger groups in our study population. The differences in the risk of hospital admission associated with the use of these 2 drugs remained significant independent of the specific comorbidities reported by the 2 sets of drug recipients.

The results of clinical trials, most notably MOVe-OUT [10], revealed that early use of molnupiravir (ie, within 5 days of symptom onset) reduced the RR of hospitalization or death by 30% among unvaccinated patients at mild to moderate risk for severe disease. Similarly, the EPIC-HR trial reported an 88% relative reduction of risk of hospitalization or death in patients treated with nirmatrelvir/ritonavir in 90.3% of the vaccinated population (3 doses) [11]. While both antivirals significantly reduced viral loads more effectively than placebo [10, 11], the results presented in both trials revealed that the number needed to treat was higher for molnupiravir than nirmatrelvir/ritonavir, suggesting that the latter drug may have a more robust antiviral effect [17, 20, 21].

One previously published real-world effectiveness study conducted in Hong Kong reported a lower risk of death (hazard ratio [HR], 0.76) among molnupiravir recipients than nonrecipients, although this drug had no impact on the risk of hospitalization [16]. In the same study, nirmatrelvir/ritonavir recipients exhibited a reduced risk of death (HR, 0.34) and hospitalization (HR, 0.76), with mortality risks that were consistently lower among elderly patients and in response to early antiviral use [16]. In a study conducted among US veterans, molnupiravir use reduced the 30-day risk of hospitalization or death among individuals >65 years old (RR, 0.67) [19]. Similarly, nirmatrelvir/ritonavir recipients exhibited a reduced risk for hospitalization or death (RR, 0.53), an effect that was observed primarily in those >65 years old (RR, 0.46) [19]. Notably, while no differences were observed in the RR of hospitalization and death between the molnupiravir- and nirmatrelvir/ritonavir-treated cohorts, a significant reduction in absolute risk of death was observed among the nirmatrelvir/ritonavir recipients vs those treated with molnupiravir [19]. Similarly, Wai et al [18] reported that both antivirals were associated with a reduced risk of hospital admission and mortality from all causes. While no significant differences were observed between the drugs, nirmatrelvir/ritonavir use had a stronger impact on hospital admissions. In a study by Yip et al [22], only nirmatrelvir/ritonavir was associated with a reduced HR (0.79) for hospitalization; no statistically significant differences were observed in the risk of hospitalization vs nonrecipients.

Results from clinical trials and observational studies revealed that molnupiravir and nirmatrelvir/ritonavir were associated with a reduced risk of hospitalization and death, with a more profound effect observed among elderly patients [16, 19]. No consistent differences in the effectiveness of these 2 drugs have been observed [16, 19], including one study in which a reduced absolute risk of death from COVID-19 was observed. Our results are consistent with those presented in previous publications, notably those focused on more substantial drug-associated reductions in risks of hospitalization and death among the elderly. Our findings document a reduced risk for hospitalization and death in response to nirmatrelvir/ritonavir vs molnupiravir treatment, and these are consistent with those demonstrating that the reduced risk for hospitalization was more profound among those treated with nirmatrelvir/ritonavir [18].

Our study has some limitations, the most important of which was the lack of data on comorbidities among those in the nonrecipient cohorts. To reduce the potential impact of this limitation, we focused on drug recipients who were ≥65 years old with age-matched nonrecipient controls. Given that the most frequent comorbidities—for example, cardiovascular disease, chronic pulmonary diseases, and diabetes—all increase with age, the selection of subjects in this age cohort increases the probability that the controls will exhibit comorbidities that are similar to those of the drug recipients. Another limitation is that viral subvariants contributing to the SARS-CoV-2 infections featured in this study were not typed. However, genomic surveillance studies document that the BA.1*, BA.2*, and BA.5* subvariants successively dominated during our study of molnupiravir and nirmatrelvir/ritonavir use [23].

In conclusion, our results suggest that antiviral use significantly reduces the risk of hospitalization and death from COVID-19 among outpatients at high risk during the period of BA.1*, BA.2, and BA.5* circulation in Greece. Although the risk of disease progression and death has been significantly reduced by mass vaccination, hybrid immunity, and the intrinsic characteristics of the Omicron (BA.1.1.529) subvariants, the results from our real-world study suggest that antivirals can further reduce the risk of hospitalization and death most notably in highly vulnerable populations. The use of oral antiviral therapy in these patients is thus strongly indicated to reduce the burden of disease.

## Supplementary Data

Supplementary materials are available at *The Journal of Infectious Diseases* online. Consisting of data provided by the authors to benefit the reader, the posted materials are not copyedited and are the sole responsibility of the authors, so questions or comments should be addressed to the corresponding author.

## Supplementary Material

jiad324_Supplementary_Data

## Notes

***Author contributions.*** Conceptualization: D. P., E. M., T. Z., M. G., S. T., A. A. Investigation–sample collection: M. Gk., K. M., G. G., N. P., K. G., E. G. K., S. L., A. K., A. S., E. T., G. S. Formal analysis: M. Gk., E. G. K., D. Z., D. F. Writing–original draft preparation: D. P., E. G. K., E. M., S. T., A. A. Writing–review and editing: M. Gk, M. G., K. M., G. G., N. P., K. G., S. L., A. K., A. S., E. T., G. S., D. Z., D. F., T. Z. Supervision: D. P., S. T., A. A. All authors have read and agreed to the published version of the manuscript. Data are not publicly available.

***Financial support*.** The publication cost was supported by the National Public Health Organization, Greece.
