# Supplementary material for: Real-world Effectiveness of Molnupiravir and Nirmatrelvir/Ritonavir as Treatments for COVID-19 in Patients at High Risk
Source: J Infect Dis. 2023 Aug 11;228(12):1667–74. doi: 10.1093/infdis/jiad324 (PMC10733724; doi:10.1093/infdis/jiad324)
Supplement: jiad324_Supplementary_Data [file jiad324_supplementary_data.zip › Supplementary data.docx]

**Supplementary data**

**Materials and Methods**

**Study population**

Antivirals were available and dispensed to the public through hospital pharmacies and were distributed by a free courier service. Eligibility and prescribing requirements were set by the national guidelines for SARS-CoV-2 antiviral treatment. Patients were eligible to receive oral antivirals if they had a positive nucleic acid amplification or rapid antigen test and had at least one risk factor for progression to severe disease. This included patients who were immunocompromised secondary to a disease or medication (including human immunodeficiency virus-positive individuals with < 200 CD4^+^ T cells), on hemodialysis, diagnosed with cystic fibrosis, age ≥ 65 years (for molnupiravir) or age ≥ 75 years (for nirmatrelvir/ritonavir). Other risk factors included age ≥ 60 years with at least one chronic comorbidity or age < 60 years with at least two chronic comorbidities. Physicians identified eligible patients who were within three days of the onset of symptoms or positive test results and referred them for oral antiviral treatment via an electronic platform that incorporated the aforementioned eligibility criteria. Medication adherence was assessed by asking patients to determine the number of pills missed within the five-day treatment period. The questionnaire included the following options: (i) none, (ii) 0 < x ≤ 5, (iii) 5< x ≤ 12, (iv) <12 x < all, or (v) all. Patients were classified into two categories based on their responses to this questionnaire. Patients categorized as poorly adherent were those with all or > 12 pills missed, corresponding to two missed daily doses for both drugs. Patients categorized as completely adherent were those who missed no pills. The categories (ii) and (iii) were not included in the analysis. Adverse drug reactions were reported during a 28-day follow-up period after the prescription of antivirals.

Demographic data, time of infection, history of a previous SARS-CoV-2 infection, and vaccination status were available from the COVID-19 national registry and SARS-CoV-2 surveillance data maintained by the National Public Health Organization (NPHO) in Greece.

As noted above, patients ≥65 years of age were included in our analysis; 4,240 of these individuals received molnupiravir and 13,861 received nirmatrelvir/ritonavir. BA.1* was the dominant circulating subgroup of the Omicron variants until end of February 2022; after this time, BA.2* became the most prevalent subgroup and remained dominant until the end of May 2022. The BA.2* subgroup was gradually replaced by the BA.5* subgroup [1]. Thus, Omicron BA.1* and BA.2* were the predominant subgroups in circulation during the period of molnupiravir use, while BA.5* dominated beginning in June 2022. Similarly, while the BA.1* and BA.2* subvariants were in circulation during the early months of our analysis of patients prescribed nirmatrelvir/ritonavir, the virus surge observed during June and July of 2022 was due to the BA.5* subvariant (<http://www.eody.gov.gr>).

**Study design**

We designed a matched retrospective cohort study to estimate the effectiveness of molnupiravir and nirmatrelvir/ritonavir in preventing hospital admission or death in patients infected with SARS-CoV-2. We identified all outpatient molnupiravir recipients who were 65 years of age or older (n = 4,240) during the period noted above; this included 65.5% of all patients who were prescribed molnupiravir during this period (February 2 through March 5, 2022). We also identified 13,861 nirmatrelvir/ritonavir recipients, representing 59.8% of all patients treated with this drug combination during the subsequent period (March 26 through July 20, 2022). Because we had no access to information on comorbidities in the non-recipient controls, we limited our analysis to those ≥ 65 years of age. Given that comorbidities typically increase with age, age-matched non-recipient controls are more likely to have a similar pattern of comorbidities as those receiving antiviral treatment. Outpatient oral antiviral users and non-recipient controls were matched for age and calendar week of SARS-CoV-2 diagnosis (within the same ISO week). The matching was performed separately for molnupiravir and nirmatrelvir/ritonavir recipients.

**Statistical analysis**

The relative effectiveness of each antiviral preparation was estimated for nirmatrelvir/ritonavir *versus* molnupiravir recipients adjusted for age, previous SARS-CoV-2 infection, vaccination status, the time elapsed since the most recent vaccination, and comorbidities (i.e., obesity, i.e., body mass index [BMI] ≥ 35 kg/m^2^, cardiovascular disease, type 2 diabetes mellitus [T2DM], and chronic liver, kidney, or lung disease). The comorbidities were included in the model as a four-level independent variable (i.e., one, two, three, or more than three comorbidities) after the exclusion of individuals with moderate to severe immunodeficiency. The effect of treatment adherence was evaluated for all drug recipients by comparing patients with complete *versus* poor or incomplete adherence, adjusted for age, previous SARS-CoV-2 infection, vaccination status, and time elapsed since the most recent vaccination.

We defined drug effectiveness as the inverse of the odds ratio (OR) for symptomatic disease, death, or the combined outcome for patients taking molnupiravir or nirmatrelvir/ritonavir *versus* outcomes determined for the non-recipient controls. The findings are reported as ORs and 95% confidence intervals (CIs) with the level of significance (*p*) set at 0.05. All analyses were conducted using R software.

**Results**

**Characteristics of the study population**

Forty percent of the patients were 80 years of age or older (40.87% of the total). Further evaluation revealed that 78.3% and 0.1% had received one and two booster doses, respectively, and 6.8% had completed the basic vaccination scheme. Of note, most of the study participants had been vaccinated relatively recently (≤ 6 months) when they contracted SARS-CoV-2 infection (Table 1). The vaccination status and the time elapsed since the previous vaccination were almost identical when comparing drug recipients and non-recipient controls (Table 1).

In the cohort of nirmatrelvir/ritonavir recipients, 68.1% and 16.6% had received one and two booster doses, respectively; these proportions are similar to those reported among the non-recipients (Table 1). However, patients treated with nirmatrelvir/ritonavir received a first booster shot somewhat earlier than those treated with molnupiravir; many (42.6%) had received a single booster dose more than 6 months before the time of infection (Table 1). Among the nirmatrelvir/ritonavir recipients, 2.14% (n = 297), 0.1% (n =14), and 0.63% (n = 88) developed symptoms, severe disease, or died from COVID-19, respectively. The percentages of patients that developed symptomatic disease (6.18%; n = 857) and ultimately died (1.87%; n = 259) were higher among the matched non-recipients (Table 1).

**Comorbidities**

The most frequent comorbidity for molnupiravir recipients is cardiovascular disease (46.3%), followed by T2DM (19.1%) and obesity (10.3%). With respect to drug adherence, 75.6% of the study participants reported complete adherence (no pills missed), while 8.3% and 2.2% reported ≤ 5 pills and ≤ 12 pills missed, respectively (Table 2). Moreover, 4.1% and 9.8% of the participants reported >12 pills and all pills missed (i.e., poor adherence) (Table 2). Adverse drug reactions were reported by 3.8% of the molnupiravir recipients, while 94.7% reported no drug reactions whatsoever (Supplementary Table 1). Gastrointestinal effects, allergy, headache/dizziness, and other adverse events were reported by 2.52%, 0.17%, 0.52%, and 0.61% of the participants, respectively (Supplementary Table 1).

**Relative effectiveness of nirmatrelvir/ritonavir versus molnupiravir and the effect of comorbidities**

Our statistical analysis revealed that patients treated with nirmatrelvir/ritonavir had a lower relative risk for developing symptomatic disease compared to those treated with molnupiravir (OR = 0.58, *p* < 0.001), adjusted for age, previous SARS-CoV-2 infection, vaccination status, and comorbidities (model 1, Table 5). As in the previous models, vaccination and previous SARS-CoV-2 infection reduced the relative risk of hospitalization, while increasing age and the number of comorbidities had the opposite effect (model 1, Table 5). The risk of hospitalization increased with the number of comorbidities; patients reporting two or more comorbidities exhibited a higher risk of hospitalization (model 1, Table 5) compared to those with no comorbidities. Comorbidities were associated with a higher risk of hospitalization only among elderly patients (model 6, Table 5). Comorbidities were excluded from the final analysis of those between the ages of 65–79 years because these factors had no association with the outcome (Table 5). Interestingly, the reduced relative risk of death for those treated with nirmatrelvir/ritonavir trended toward statistical significance (OR = 0.69, *p* = 0.09) (model 2, Table 5). A similar effect was observed when considering vaccination status, comorbidities, and age, although no significant effect was observed based on previous SARS-CoV-2 infections. Interestingly, in a subsequent set of analyses focused on specific age groups, the two drugs exhibited significantly different degrees of effectiveness which were most pronounced in younger age cohorts (ages 65–69 years, OR = 0.30, *p* = 0.002; ages 70–74 years, OR = 0.39, *p* = 0.003; age ≥80 years, OR = 0.69, *p* = 0.03; models 3, 4, and 6, Table 5). No difference in relative effectiveness was observed among individuals who were between 75–79 years of age (model 5, Table 5).

**References**

1. Kopsidas I, Karagiannidou S, Kostaki EG, et al. Global Distribution, Dispersal Patterns, and Trend of Several Omicron Subvariants of SARS-CoV-2 across the Globe. Trop Med Infect Dis **2022**; 7.
