# Supplementary material for: Real-world Effectiveness of Molnupiravir and Nirmatrelvir/Ritonavir as Treatments for COVID-19 in Patients at High Risk
Source: J Infect Dis. 2023 Aug 11;228(12):1667–74. doi: 10.1093/infdis/jiad324 (PMC10733724; doi:10.1093/infdis/jiad324)
Supplement: jiad324_Supplementary_Data [file jiad324_supplementary_data.zip › Supplementary Table1.docx]

|  | **Molnupiravir**  **recipients** | | **Νirmatrelvir/ritonavir recipients** | |
| --- | --- | --- | --- | --- |
| **Adverse drug reactions** | **Ν** | **%** | **Ν** | **%** |
| Gastrointestinal (GI) effects | 107 | 2.52% | 143 | 1.03% |
| Allergy | 7 | 0.17% | 3 | 0.02% |
| Headache, dizziness | 22 | 0.52% | 11 | 0.08% |
| Other | 21 | 0.61% | 28 | 0.20% |
| Subtotal | 162 | 3.82% | 185 | 1.33% |
|  |  |  |  |  |
| No adverse drug reactions | 4013 | 94.65% | 3274 | 23.62% |
| Unknown | 65 | 1.53% | 10402 | 75.05% |
|  |  |  |  |  |
| Total | 4240 | 100% | 13861 | 100.0% |

**Supplementary Table 1 Adverse drug reactions for molnupiravir and nirmatrelvir/ritonavir recipients**
