# Supplementary material for: Real-world Effectiveness of Molnupiravir and Nirmatrelvir/Ritonavir as Treatments for COVID-19 in Patients at High Risk
Source: J Infect Dis. 2023 Aug 11;228(12):1667–74. doi: 10.1093/infdis/jiad324 (PMC10733724; doi:10.1093/infdis/jiad324)
Supplement: jiad324_Supplementary_Data [file jiad324_supplementary_data.zip › Supplementary Table2.docx]

**Supplementary Table 2 Multivariable logistic regression analysis for the effectiveness of molnupiravir treatment**

| **Explanatory variable** | **Odds Ratio** | **95% CI^b^** | **p-value** |
| --- | --- | --- | --- |
| **Model 1**  **Hospital admission including ICU^a^, clinical deterioration or death *versus* no hospitalization, ICU admission, clinical deterioration, or death** |  |  |  |
| **Treatment** |  |  |  |
| Non-recipients | 1.00 | - | - |
| Molnupiravir recipients | 0.40 | 0.34-0.48 | <0.001 |
| **Age (in years)** | 1.07 | 1.06-1.08 | <0.001 |
| **Previous infection with COVID-19** |  |  |  |
| No previous SARS-CoV-2 infection | 1.00 | - | - |
| Previous SARS-CoV-2 infection | 0.45 | 0.19-0.92 | 0.02 |
| **Vaccination status** |  |  |  |
| Unvaccinated | 1.00 | - | - |
| Vaccination (2, 3, or 4 doses ≤ 6 months before index SARS-CoV-2 infection) | 0.34 | 0.29-0.41 | <0.001 |
| Vaccination (2, 3, or 4 doses > 6 months before index SARS-CoV-2 infection) | 1.17 | 0.75-1.77 | 0.48 |
| **Model 2**  **Hospitalization without ICU admission, clinical deterioration, or death *versus* no hospitalization, ICU admission, clinical deterioration, or death among those 65**–**69 years of age** |  |  |  |
| **Treatment** |  |  |  |
| Non-recipients | 1.00 | - | - |
| Molnupiravir recipients | 1.05 | 0.60–1.82 | 0.87 |
| **Previous COVID-19** |  |  |  |
| No previous SARS-CoV-2 infection | 1.00 | - | - |
| Previous SARS-CoV-2 infection | 0.47 | 0.07–1.66 | 0.31 |
| **Vaccination status** |  |  |  |
| Unvaccinated | 1.00 | - | - |
| Vaccination (2, 3, or 4 doses ≤ 6 months before index SARS-CoV-2 infection) | 0.19 | 0.11–0.35 | <0.001 |
| Vaccination (2, 3, or 4 doses > 6 months before index SARS-CoV-2 infection) | 0.77 | 0.11–2.83 | 0.73 |
| **Model 3a**  **Hospitalization without ICU admission, clinical deterioration, or death *versus* no hospitalization, ICU admission, clinical deterioration, or death among those 70**–**74 years of age** |  |  |  |
| **Treatment** |  |  |  |
| Non-recipients | 1.00 | - | - |
| Molnupiravir recipients | 0. 65 | 0.38–1.08 | 0.10 |
| **Previous COVID-19** |  |  |  |
| No previous SARS-CoV-2 infection | 1.00 | - | - |
| Previous SARS-CoV-2 infection | 0.45 | 0.02–2.32 | 0.44 |
| **Vaccination status** |  |  |  |
| Unvaccinated | 1.00 | - | - |
| Vaccination (2, 3, or 4 doses ≤ 6 months before index SARS-CoV-2 infection) | 0.41 | 0.23–0.75 | 0.003 |
| Vaccination (2, 3, or 4 doses > 6 months before index SARS-CoV-2 infection) | 2.98 | 1.00–7.94 | 0.04 |
| **Model 3b**  **Hospitalization without ICU admission, clinical deterioration, or death *versus* no hospitalization, ICU admission, clinical deterioration, or death among those 70–74 years of age** |  |  |  |
| **Treatment** |  |  |  |
| Non-recipients | 1.00 | - | - |
| Molnupiravir recipients | 0. 63 | 0.39–1.04 | 0.07 |
| **Vaccination status** |  |  |  |
| Unvaccinated | 1.00 | - | - |
| Vaccinated (2, 3, or 4 doses) | 0.49 | 0.28–0.87 | 0.01 |
| **Model 4**  **Hospitalization without ICU admission, clinical deterioration, or death *versus* no hospitalization, ICU admission, clinical deterioration, or death among those 75**–**79 years of age** |  |  |  |
| **Treatment** |  |  |  |
| Non-recipients | 1.00 | - | - |
| Molnupiravir recipients | 0. 42 | 0.26–0.68 | <0.001 |
| **Vaccination status** |  |  |  |
| Unvaccinated | 1.00 | - | - |
| Vaccination (2, 3, or 4 doses ≤ 6 months before index SARS-CoV-2 infection) | 0.32 | 0.20–0.54 | <0.001 |
| Vaccination (2, 3, or 4 doses > 6 months before index SARS-CoV-2 infection) | 0.85 | 0.13–3.25 | 0.84 |
| **Model 5**  **Hospitalization without ICU admission, clinical deterioration, or death *versus* no hospitalization, ICU admission, clinical deterioration, or death among those 80 years of age and older** |  |  |  |
| **Treatment** |  |  |  |
| Non-recipients | 1.00 | - | - |
| Molnupiravir recipients | 0.29 | 0.22–0.39 | <0.001 |
| **Vaccination status** |  |  |  |
| Unvaccinated | 1.00 | - | - |
| Vaccination (2, 3, or 4 doses ≤ 6 months before index SARS-CoV-2 infection) | 0.43 | 0.33–0.60 | <0.001 |
| Vaccination (2, 3, or 4 doses > 6 months before index SARS-CoV-2 infection) | 0.91 | 0.42–1.79 | 0.80 |

^a^ICU, intensive care unit, ^b^CI, confidence interval
