# Supplementary material for: Real-world Effectiveness of Molnupiravir and Nirmatrelvir/Ritonavir as Treatments for COVID-19 in Patients at High Risk
Source: J Infect Dis. 2023 Aug 11;228(12):1667–74. doi: 10.1093/infdis/jiad324 (PMC10733724; doi:10.1093/infdis/jiad324)
Supplement: jiad324_Supplementary_Data [file jiad324_supplementary_data.zip › Supplementary Table3.docx]

**Supplementary Table 3. Multivariable logistic regression analysis to determine the effectiveness of treatment in the molnupiravir recipients with different levels of adherence**

| **Explanatory variable** | **Odds Ratio** | **95% CI^a^** | **p-value** |
| --- | --- | --- | --- |
| **Model 1**  **Hospitalization with no ICU^b^ admission, clinical deterioration, or death *versus* no hospitalization, ICU admission, clinical deterioration, or death** |  |  |  |
| **Age (in years)** | 1.02 | 1.00-1.05 | 0.02 |
| **Previous infection with COVID-19** |  |  |  |
| Non-previous SARS-CoV-2 infection | 1.00 | - | - |
| Previous SARS-CoV-2 infection | 0.21 | 0.01-1.00 | 0.13 |
| **Vaccination status** |  |  |  |
| Unvaccinated | 1.00 | - | - |
| Vaccination (2 or 3 or 4 dose <= 6 months) | 0.26 | 0.17-0.38 | <0.001 |
| Vaccination (2 or 3 or 4 dose > 6 months) | 0.30 | 0.05-1.02 | 0.10 |
| **Drug adherence^c^** |  |  |  |
| Partial or poor adherence | 1.00 | - | - |
| Total adherence | 0.27 | 0.18-0.39 | <0.001 |
| **Model 2**  **Death with or without ICU admission or clinical deterioration *versus* hospitalization, ICU admission, clinical deterioration or, death** |  |  |  |
| **Age (in years)** | 1.04 | 1.01-1.08 | 0.02 |
| **Previous infection with COVID-19** |  |  |  |
| Non-previous SARS-CoV-2 infection | 1.00 | - | - |
| Previous SARS-CoV-2 infection | 0.65 | 0.04-3.41 | 0.68 |
| **Vaccination status** |  |  |  |
| Unvaccinated | 1.00 | - | - |
| Vaccination (2 or 3 or 4 dose <= 6 months) | 0.24 | 0.12-0.47 | <0.001 |
| Vaccination (2 or 3 or 4 dose > 6 months) | 0.46 | 0.03-2.40 | 0.46 |
| **Drug adherence** |  |  |  |
| Partial or poor adherence | 1.00 | - | - |
| Total adherence | 0.24 | 0.13-0.45 | <0.001 |

^a^CI, Confidence Interval; ^b^ICU, Intensive Care Unit; ^c^Partial or poor adherence included patients with ≥ 12 pills missed, and complete adherence those with no pills missed
