# Supplementary material for: Real-world Effectiveness of Molnupiravir and Nirmatrelvir/Ritonavir as Treatments for COVID-19 in Patients at High Risk
Source: J Infect Dis. 2023 Aug 11;228(12):1667–74. doi: 10.1093/infdis/jiad324 (PMC10733724; doi:10.1093/infdis/jiad324)
Supplement: jiad324_Supplementary_Data [file jiad324_supplementary_data.zip › Supplementary Table4.docx]

**Supplementary Table 4 Multivariable logistic regression analysis for the effectiveness of nirmatrelvir/ritonavir treatment**

| **Explanatory variable** | **Odds Ratio** | **95% CI^b^** | **p-value** |
| --- | --- | --- | --- |
| **Model 1** |  |  |  |
| **Hospital admission including ICU^a^, clinical deterioration or death *versus* no hospitalization, ICU admission, clinical deterioration, or death** |  |  |  |
| **Treatment** |  |  |  |
| Molnupiravir non-recipients | 1.00 | - | - |
| Molnupiravir recipients | 0.32 | 0.28-0.36 | <0.001 |
| **Age (in years)** | 1.10 | 1.10-1.11 | <0.001 |
| **Previous infection with COVID-19** |  |  |  |
| Non-previous SARS-CoV-2 infection | 1.00 | - | - |
| Previous SARS-CoV-2 infection | 0.47 | 0.33-0.66 | <0.001 |
| **Vaccination status** |  |  |  |
| Unvaccinated | 1.00 | - | - |
| Vaccination (2, 3, or 4 doses ≤ 6 months before index SARS-CoV-2 infection) | 0.49 | 0.42-0.57 | <0.001 |
| Vaccination (2, 3, or 4 doses > 6 months before index SARS-CoV-2 infection) | 0.41 | 0.35-0.48 | <0.001 |
| **Model 2**  **Hospitalization without ICU admission, clinical deterioration, or death *versus* no hospitalization, ICU admission, clinical deterioration, or death among those 65**–**69 years of age** |  |  |  |
| **Treatment** |  |  |  |
| Non-recipients | 1.00 | - | - |
| Nirmatrelvir/ritonavir recipients | 0.71 | 0.44–1.14 | 0.16 |
| **Previous COVID-19** |  |  |  |
| No previous SARS-CoV-2 infection | 1.00 | - | - |
| Previous SARS-CoV-2 infection | 0.37 | 0.06–1.35 | 0.19 |
| **Vaccination status** |  |  |  |
| Unvaccinated | 1.00 | - | - |
| Vaccination (2, 3, or 4 doses ≤ 6 months before index SARS-CoV-2 infection) | 0.49 | 0.25–0.99 | 0.04 |
| Vaccination (2, 3, or 4 doses > 6 months before index SARS-CoV-2 infection) | 0.53 | 0.28–1.06 | 0.06 |
| **Model 3**  **Hospitalization without ICU admission, clinical deterioration, or death *versus* no hospitalization, ICU admission, clinical deterioration, or death among those 70**–**74 years of age** |  |  |  |
| **Treatment** |  |  |  |
| Non-recipients | 1.00 | - | - |
| Nirmatrelvir/ritonavir recipients | 0.56 | 0.39–0.79 | 0.001 |
| **Previous COVID-19** |  |  |  |
| No previous SARS-CoV-2 infection | 1.00 | - | - |
| Previous SARS-CoV-2 infection | 0.41 | 0.12–1.07 | 0.10 |
| **Vaccination status** |  |  |  |
| Unvaccinated | 1.00 | - | - |
| Vaccination (2, 3, or 4 doses ≤ 6 months before index SARS-CoV-2 infection) | 0.43 | 0.27–0.69 | <0.001 |
| Vaccination (2, 3, or 4 doses > 6 months before index SARS-CoV-2 infection) | 0.41 | 0.26–0.66 | <0.001 |
| **Model 4**  **Hospitalization without ICU admission, clinical deterioration, or death *versus* no hospitalization, ICU admission, clinical deterioration, or death among those 75**–**79 years of age** |  |  |  |
| **Treatment** |  |  |  |
| Non-recipients | 1.00 | - | - |
| Nirmatrelvir/ritonavir recipients | 0. 39 | 0.28–0.53 | <0.001 |
| **Previous COVID-19** |  |  |  |
| No previous SARS-CoV-2 infection | 1.00 | - | - |
| Previous SARS-CoV-2 infection | 0.91 | 0.38–1.94 | 0.81 |
| **Vaccination status** |  |  |  |
| Unvaccinated | 1.00 | - | - |
| Vaccination (2, 3, or 4 doses ≤ 6 months before index SARS-CoV-2 infection) | 0.53 | 0.34–0.82 | 0.004 |
| Vaccination (2, 3, or 4 doses > 6 months before index SARS-CoV-2 infection) | 0.47 | 0.31–0.73 | <0.001 |
| **Model 5**  **Hospitalization without ICU admission, clinical deterioration, or death *versus* no hospitalization, ICU admission, clinical deterioration, or death among those ≥80 years of age** |  |  |  |
| **Treatment** |  |  |  |
| Non-recipients | 1.00 | - | - |
| Nirmatrelvir/ritonavir recipients | 0.26 | 0.22–0.31 | <0.001 |
| **Previous COVID-19** |  |  |  |
| No previous SARS-CoV-2 infection | 1.00 | - | - |
| Previous SARS-CoV-2 infection | 0.52 | 0.30–0.84 | 0.01 |
| **Vaccination status** |  |  |  |
| Unvaccinated | 1.00 | - | - |
| Vaccination (2, 3, or 4 doses ≤ 6 months before index SARS-CoV-2 infection) | 0.54 | 0.44–0.68 | <0.001 |
| Vaccination (2, 3, or 4 doses > 6 months before index SARS-CoV-2 infection) | 0.47 | 0.38–0.59 | <0.001 |

^a^ICU, intensive care unit, ^b^CI, confidence interval
